# The Actual Process of Nutrition Demonstrated by the Microscope

**Published:** 1845-07

**Authors:** 


**THE ACTUAL PROCESS OF NUTRITION IN THE LIVING STRUCTURE DEMONSTRATED BY THE MICROSCOPE.** Second and Third Series of Experimental Researches. By *William Addison*, F.L.S., Member of the Royal College of Surgeons in London, and Surgeon to H. R. H. the Duchess of Kent. Malvern, 8vo. London, Churchill, 1843—5.

MR ADDISON has made many excellent observations respecting several constituent parts of the blood, for which, and especially considering that his locale is not favourable to scientific pursuits, he merits high commendation. This gentleman has likewise contributed something to our knowledge of the minute structure of the lungs; though, as we stated in our last number, when noticing Dr. Carpenter's Physiology, it is certain he has fallen into some most serious errors in his account of those organs, and more particularly by attributing the formation of the air-cells to the pressure exerted in the first respirations by the air in the ultimate divisions of the bronchi. Mr. Addison may be assured that, in the construction of organized bodies, Nature never proceeds upon such bare mechanical principles; a fact which must never be neglected if it be wished to penetrate to the general laws regulating the development of animal and vegetable structures.

In the treatises before us the author ascends to higher ground; indeed, if that which the title-page indicates were actually realized, the discovery, namely, of the law of nutrition, Mr. Addison would have accomplished for the science of organic life, what the law of definite proportion has effected for chemistry, and that of gravitation for matter generally, and might therefore claim a place by the side of Newton and Dalton. But such is not the case. We say this in no unfriendly spirit, or as implying any censure beyond what must attach to an undue indulgence in hypothesis; for no physiologist, who knows the amount of exact information we possess, and who is unbiassed by partial views, will hesitate in giving expression to his opinion, that the time has not yet arrived for the enunciation of the principle of nutrition. And this being so, it is merely an encumbrance to have theories framed, which must be cleared away, lest they should, by misleading, impede, as in similar cases they always have done, the progress of science.

We may add that the present is rather an inauspicious time for the appearance of a first, second, and third series of "Researches" upon the recondite matters here treated of; for cautious observers have had their faith in such transcendental speculations, not a little shaken by a somewhat similar series respecting the metamorphoses of the red corpuscles and the hypothesis of the double spiral of Dr. M. Barry.

In making this assertion we in no degree participate in the somewhat ungenerous attempts which have been made to depreciate the high merits of the last-named eminent physiologist as an original and successful cultivator of embryology: we wish simply to express our conviction of the evils which must result from attempting to establish general laws upon such totally insufficient grounds as we find in these "Researches." Many of our readers are doubtless acquainted with the leading views of Mr. Addison, as these have from time to time appeared in the weekly and other periodicals: this circumstance will render any lengthened notice in this place unnecessary.

The author contends that both the fibrin and the serum of the blood are contained, during the circulation, in the colourless, or lymph corpuscles; that when blood is drawn from its containing vessels, the colourless corpuscles, "from the sudden change of temperature to which they are exposed, or from some other causes, burst and discharge their contents, consisting of liquor sanguinis and molecules;" and that the fibrin then assuming a solid form, "very shortly fibrillates," and so effects the coagulation.

"What, then, it may be asked, is the nature of the liquid in which the corpuscles move when in the living vessels? It is impossible to determine: for it cannot be procured for experimental examination without being mingled with corpuscles, nor, according to these views, without some of them bursting and mingling with it their own contents." P. 8, *Second Series.*

That the colourless particles form an important part of the blood; that they are cells containing some particular matter; that they are greatly accumulated during the inflammatory condition, and consequently where the fibrin is augmented in quantity; and that they become frequently ruptured after their removal from the blood-vessels, is abundantly proved in these researches, and by those of other observers. But we do not conceive that the position, namely—that the fibrin and serum are, during the circulation, contained in the above-named corpuscles, is in any degree proved as a fact. Such an opinion is opposed to the generally-received views, and especially to those of a most careful microscopist, Mr. Wharton Jones, who, it is well known, contends that fibrin is elaborated by the metamorphosing powers of the *red*, and not of the *colourless* particles.

Mr. Addison has discovered very interesting movements of the minute molecules contained in the colourless corpuscles of the blood, and also in corpuscles found in various other fluids—as that of inflamed pimples, of the vesicles of shingles, &c. The following is an account of these motions as seen in a drop of blood taken from the leg of a young woman suffering from Erythema Nodosum.

"I found in it nearly as many colourless-cells as red ones. The colourless-cells were uniformly molecular, smaller than the mucous-cells in saliva; but, like them, filled with a number of minute dark objects or molecules. I added a drop of water at the temperature of 90, having one drop of liquor potassæ to the ounce. The colourless-cells gradually increased in size; and in the course of from three to five minutes, by very careful attention, I could see, in the majority of them, very active motions among the contained molecules. I also saw some of them open and discharge myriads of minute active molecules, without any immediate alteration in their figure or appearance. The motions of the molecules remaining in the interior of the cells, however, gradually ceased after this event, and the cells slowly changed, showing large coarse granules, or discs." P. 3, *Third Series*.

Motions similar to those here described have been noticed by other, and some of them earlier, observers. Thus Schwann saw them among the granules contained in the cells of the germinal membrane of the hen's egg; and Dr. Todd and Mr. Bowman state, in their "Physiological Anatomy," (p. 60,) that a molecular motion of the same kind may be seen in the very minute granules, which occupy the cells of the membrana pigmenti of the choroid coat. We have ourselves seen very active motions of the same kind in cells taken from a malignant growth of the penis, where they were first noticed by an intelligent friend, and by him pointed out to the writer. It is difficult to assign the true cause of these remarkable movements, which offer a most interesting microscopic phenomenon. Brown's molecular motion occurring equally, as it does, in extremely minute particles of matter, whether organic or inorganic, is evidently not of a vital character; in fact, it is known to depend upon the evaporation of the fluid, in which it is requisite, in order to produce the movements, the particles should be immersed. In the case before us the corpuscles in which the phenomenon takes place, are exposed in the manipulation necessary to prepare the object for the microscope to the influence of causes, such as evaporation, and, in some cases, to endosmose, which are in themselves efficient to produce the movements under consideration; and it is to these physical causes, which, in the case of molecules varying from the $\frac{1}{5000}$ to the $\frac{1}{15000}$ and $\frac{1}{24000}$ of an inch, are all powerful, we are inclined to attribute these motions rather than to any vital influence. The notion of Dr. Houston, apparently adopted by the author, that the molecules are self-active, and perform "voluntary evolutions," we hold to be totally fallacious.

A large part of these researches consists of experiments to prove the identity of mucous and pus globules with the colourless corpuscles of blood; or rather, we might say, to shew that these said corpuscles, called by the author "nutritive particles," are the common source of all parts of the body—fibrous tissue, mucus, saliva and "other secretions," and epithelia; and, as these wonder-working corpuscles, also, elaborate and contain all the fibrin and albumen of the blood, also of muscle, nerve and so forth. This is one of those sweeping generalizations, springing from a favourite hypothesis, which have become rife in late years owing to the novelty and interest attaching to the use of the powerful and excellent microscopes now constructed. All we can say is, that, after going through these two treatises, we hold most of the positions to be "non proven;" many of them rest only upon hypothesis; and of a few only can it be predicated, that they are supported by sufficient facts to give them some reasonable ground of probability. Mr. Addison finds that, if equal parts of pus and liquor potassæ be well mingled together, they form "a transparent and exceedingly plastic compound—mucus or tissue." (3rd Series, p. 17.) Further, that in this way is produced "a most delicate, thin, transparent and highly elastic fibrous membrane, exactly resembling some of the thin transparent membranes of the embryo, except in the presence of blood-vessels, or the structureless basement membrane of Mr. Bowman." That a film may thus be formed we doubt not, but it is by no means thence proved that it is an organized membrane; at all events, such an inference seems to be opposed to the observations of one whom we must hold to be a better authority in a question like this, Mr. J. Goodsir, who affirms that the basement membrane is the result of the aggregation and condensation of nucleated cells.

Some of Mr. Addison's experiments on inflammation have given interesting results, though the value of these, as elsewhere, is lessened by the intermixture of speculations which are not proved, and which we hold to be most improbable; such, for example, that the red corpuscles of the blood are not only detained in the inflamed vessels, but that, passing through the friable walls of the capillaries, they enter into the composition of the surrounding tissue. (Second Series, p. 59). A change is described as occurring in the blood in consequence of irritation, which is important, as showing a condition not before noticed, so far as we are aware, and which may be *one* of the causes of the obstruction that follows the application of a stimulus to the capillary vessels: we allude to a thickening and stickiness which occur in the blood under these circumstances, and which the author attributes to the accumulation of colourless cells and numerous molecules. (3rd Series, p. 83).

The main result which Mr. Addison aims at obtaining by all these multitudinous and somewhat laboured researches, is the establishment of a *theory of nutrition*. Our limits will not allow us to follow the author through all these details, and we must therefore rest satisfied with stating that he conceives the colourless corpuscles of the blood more or less modified to be the grand source of all nutritious matter; that the colourless blood-corpuscles adhere to the tissue forming the boundary of the blood-channels; that they pass into, and contribute to form the tissue (that is, the parietes of the capillaries), and are afterwards evolved or thrown off from the nearest free surface—a follicle, crypt, or duct; the epithelial scales and the mucus, or the secretions flowing from the follicles or ducts, being the result of the dissolution of the cells and tissues. We must express distinctly our conviction, that no such passage of colourless corpuscles through the walls of the capillaries ever does in reality take place. Those who have carefully studied the appearance which these vessels present when examined by the microscope will, we think, agree with us in this conviction. In whatever part of the body the capillaries are thus inspected, they present limiting walls as definitely marked as the neurilemma of the primary nervous tubules; and through walls thus disposed, it is difficult to conceive of the transmission of bodies having the size and character of the colourless corpuscles. It is likewise necessary to point out that the theory of nutrition here advocated is opposed to the views flowing from Schwann's researches into the genesis of the *nucleated cell;* at least it seems to us difficult to reconcile the exact and satisfactory investigations upon this latter process, with Mr. Addison's account of the production of the epithelium and other tissues.

In conclusion we may remark, that although the grandiloquent and Baconian style adopted by the author, and his frequent indulgence in speculation and inference which the facts adduced in no degree support, greatly deteriorate the value of these researches, they are well worthy of the attention of all who are interested in the progress of minute anatomy.

